# Why Psychotropic Drugs Don't Cure Mental Illness—But Should They?

**DOI:** 10.3389/fpsyt.2021.579566

**Published:** 2021-04-06

**Authors:** Iliyan Ivanov, Jeffrey M. Schwartz

**Affiliations:** ^1^Icahn School of Medicine at Mount Sinai, Department of Psychiatry, New York, NY, United States; ^2^Department of Psychiatry and Biobehavioral Sciences, University of California, Los Angeles, Los Angeles, CA, United States

**Keywords:** psychotropic, consciousness, computational modeling, neuroscience, psychopathology, bio-psycho-social, psychotherapeutic, language

## Abstract

While pharmacological treatments for psychiatric disorders have offered great promise and have provided clinically meaningful symptom relief these treatments have had less effect on altering the course of these disorders. Research has provided many new insights about the effects of different psychotropic agents on the functions of various brain systems as investigators have embraced the “translational research model.” However, this theoretical approach of deconstructing complex behaviors into smaller measurable behavioral units and targeting brain systems that are hypothesized to underlie these discrete behaviors has offered little of practical clinical relevance to significantly improve the treatment of psychiatric disorders in this century. Radical new treatments have not emerged, and available treatments continue to provide symptom relief without resolution of the underlying conditions. Recent publications on the subject have attempted to identify the barriers to progress and have pointed out some of the limitations of the translational approach. It is our position that, given the present limitations of our therapeutic arsenal, both researchers and clinicians would be well-advised to pay closer attention to human specific factors such as the role of language, the creation of personal narratives, and how factors such as these interface with underlying biological diatheses in mental illness. These interactions between pathophysiology and intrapersonal processes may be critical to both the *in vivo* expression of the underlying biological mechanisms of psychiatric disease states, and to the development of enhancements in therapeutic efficacy. Lastly, we discuss the implications of more coherently integrating neuroscientific research and clinical practice for more effectively addressing the challenges of understanding and treating mental illness.

## Introduction

Psychotropic drugs have been playing an increasing role in the treatment of mental disorders for over half a century; they have offered substantial relief from some debilitating symptoms like auditory hallucinations, excessive mood fluctuations, etc. We know that these drugs influence certain brain chemicals and alter gene expression and protein synthesis; however, these biological effects do not translate into lasting positive psychological effects since the symptoms tend to return when medications are stopped and recur even as patients continue to take complex regimens of antidepressants, antipsychotics, mood stabilizers, anxiolytics, and the like. Despite an ever-increasing compilation of evidence concerning the effects of drugs on various biological brain systems, the question remains—why is it so that psychotropic drugs do not cure mental disorders? And further—should we expect them to do so?

Current approaches to the understanding of neuropsychiatric disorders revolve around efforts to distill and reduce complex behaviors into measurable discrete behavioral units and to map these component behaviors onto neuronal networks. The hope is that clear description, measurements and neuronal mapping of discrete behaviors may bring better understanding of complex behaviors. In theory, this approach should allow us to manipulate the larger behavioral complex and improve symptoms, and more importantly change the behaviors in some fundamental and long-lasting fashion—in other words should allow us to “cure” (or at the very least, significantly ameliorate) mental illness.

In the context of this theoretically driven hypothesis/opinion paper our use of the term “cure” differs somewhat from its most common literal meaning. It is well-accepted that few medical therapeutic interventions result, *per se*, in a cure (with possible exceptions such as antibiotic and surgical treatments). For the majority of medical conditions current treatments result in the improvement of symptoms and in maintenance of interventions to sustain those improvements—which is often also the case in the use of psychotropic medications in psychiatry. Therefore, we use the term “cure” to draw special attention to and enhance the understanding that, as effective as they are, medications in psychiatry are really most useful when administered with a proper appreciation of the parameters of their limitations. In other words, the available psychopharmacological agents offer a great deal of symptom relief, which in turn has the potential to be of life changing importance to many patients—but they are not disease specific and often are used across diagnostic categories, their discontinuation often is associated with disease relapse, and they have shown little evidence related to their ability to change the trajectory of psychiatric disorders (e.g., medications for attention deficits/hyperactivity and autism spectrum disorders are often introduced in early development, but the developmental trajectory of these disorders does not seem to be affected by the pharmacological treatment in the sense of reduced rates of persistence in older age). In this way, one may think of them as serving a similar function to anti-inflammatory agents. However, the main premise of this paper is to suggest that medication treatments in psychiatry that are useful for the purpose of symptom control may enhance mental function in ways that critically facilitate improved communication and the ability to converse and think in more effective ways. In that way psychiatric drugs fundamentally differ from other pharmaceuticals used in medicine. This enhancement of mental function may then directly result in important changes in psychological constructs that are not in and of themselves reducible to biological mechanisms related to the mode of action of the medication itself. Some recent novel theoretical perspectives have emerged attempting to bridge the gap between the knowledge obtained from translational research and the use of language and psychological concepts (e.g., how anxiety differs from fear) and apply the resulting synergies between biological and psychological insights in an attempt to improve the development and therapeutic efficacy of psychotropic agents (1, 2).

Current approaches to understanding neuropsychiatric disorders face a multiplicity of challenges, both psychological and philosophical. One psychological challenge casts doubt on the assumption that the sum of individual parts (i.e., discrete behaviors) is sufficient to fully explain and understand the larger behavioral complex. And philosophically, the question of how these larger behavioral complexes are precisely related to the function of the brain raises a quagmire of deep, difficult, and very delicate issues. Some have proposed that psychological processes including conscious experiences that are essential in the expression of mental disorders are linked to and emerge from the complexity of brain functions. One very obvious question is raised by the fact that the more we learn about the brain, the more it appears that the majority of its functions are not related to consciousness at all. Most of the anatomically outlined neuronal pathways control bodily functions for which humans are not conscious most of the time; many of these processes actually never reach consciousness (regulation of body temperature, oxygen and carbon dioxide levels in plasma and so on), while some (e.g., breathing) have an apparently dualistic existence, at least with respect to consciousness—they can potentially be attended to and experienced as conscious (even volitional) events, but more often are not and go unnoticed. Indeed, in actuality a relatively small part of the total complex of brain seems to relate to conscious experiences. And yet mental illnesses are, to a very clinically significant degree, a set of disorders of consciousness.

The primary aim of this paper will be to discuss the challenges linked to the psychological aspect of the overview presented above. These challenges could be summed up as follows: the fact that many psychological states cannot be adequately mapped out onto distinct neuronal pathways very significantly limits any attempt to apply the translational approach in its current form as the best means for advancing progress in the development of maximally effective new biological treatments. Acknowledging that this lack of understanding of psychophysical mechanisms may represent an inherent limitation on the clinical utility of medications in psychiatry to treat clinically important psychological deficits, the optimization of treatment may be best accomplished by adopting a new paradigm (e.g., LeDoux/Pine two system model and related models) that accounts for the role of brain structures and their relationship to conscious experience and the use of language. For instance, a subject is asked to perform a test designed to measure the ability to inhibit prepotent motor response (i.e., repetitive button presses); by extension such an experiment might measure the subject's overall ability to withhold behaviors that may lead to potentially negative consequences. However, in real life the person might be perfectly capable of withholding potentially negative behaviors in the context of some particular circumstances and much less capable to do so in different circumstances; or this ability may vary as a function of subject's emotional state(s)—and so on. Further, one can also argue that the ability to control our own behaviors is strongly influenced by other mental states like “confidence.” In short, we will attempt to make the case that without better understanding of the relationship between biological (e.g., brain based) and psychological processes the idea that advances in psychopharmacology will translate into more efficient clinical care for individuals with mental illness might be too optimistic.

## Integrating Neuroscience, Psychology, and Philosophy

In this section we will review some of the current ideas that bridge the wealth of inter- related bodies of knowledge from neuroscience, psychology and philosophy. This approach has been adopted by others as it is considered helpful to examine the contribution of different paradigms to the topics of reconciling the gaps between different assessments in psychiatry and neuroscience (3). We may start with the concept of “confidence” mentioned above. It should be noted here that “confidence” as part of many psychological studies is measured by the reaction time for one's response to a task—faster responses are assumed to reflect greater confidence on the part of the subject; for the purpose of the current discussion the concept of “confidence” refers to a more complex psychological set of traits, such as self- assurance in one's own abilities, and is not considered measurable by simple reaction times. Recent work in the emerging field of philosophical neuroscience supports the deep complexity of the concept of confidence. Lau and Rosenthal (4), in a seminal review article, elaborated on frontal cortical mechanisms related to subjective reports of experience and confidence in the accuracy of those reports, and Fleming and Lau (5) proposed empirical tests to measure the relationship between these two experiential states. Subjective reports of experience, of course, comprise a core intrinsic element of all psychotherapeutic interventions, and better understanding and delineating the multiple relationships between subjective reports and confidence is potentially of great clinical significance.

Confidence may also emerge as a personality trait not necessarily linked to one's actual skills and/or may develop in result of consistent efforts to improve one's skills.

Furthermore, some cultures are more adept at promoting personal confidence, while others may suppress it. As a result, the constitutional vs. acquired sense of self-efficacy may produce very different results with respect to clinically relevant and psychotherapeutically empowering concepts of confidence, which are independent of one's actual ability to perform a lab task. In short, the discrete behavioral construct (i.e., ability to withhold a prepotent button press) may vary in its presentation within more complex behaviors depending on its interaction with other psychological states. And although the ability to withhold a motor response could possibly be mapped onto some discrete neuronal pathway, the psychological construct of clinically relevant states of confidence will most likely not be possible to link to any specifiable “confidence” networks.

The purpose of the above discussion on the different aspects of “confidence” is 2-fold: first, it illustrates that terms that can appear to be used interchangeably may carry important semantic differences in biological as opposed to psychological usage and practice. These kinds of differences in construed meaning are very theoretically and practically important with respect to a maintaining a proper perspective on the relationship between, on the one hand, discovering and understanding pathophysiological mechanisms, and on the other hand, communicating and applying this knowledge in clinical practice as effectively as possible. Second, while it is perfectly appropriate for the purpose of a study of physiological mechanisms to reduce complex psychological constructs into measurable units like “reaction time” and “accuracy,” recognizing the limitations of these measures, insofar as they do not fully specify and account for the psychological meaning of the construct, is similarly very theoretically and practically important. A major aspect of our thesis is that mental health researchers and practitioners must make a special effort to remain aware of the deep complexity of the relationships between biological and psychological terminology and concepts. Humility is called for by all concerned working at the interface of neuroscience and mental functioning.

Inferences related to the biology that underlies “confidence,” when it is measured by bio- behavioral indexes, may not be fully relevant to the mechanisms and meaning of the psychological trait of confidence, and how it may (or may not) be related to any purported brain mechanisms.

There are numerous examples of behavioral processes that can be reliably linked to the activity of particular brain regions (e.g., the receptive and expressive speech centers in the brain, motor inhibitory networks, the motivation-reward system, posterior and anterior attentional networks among others), and some of them involve mental functions of considerable complexity. For example, the present authors, in collaboration with philosopher LaRock et al. (6), have recently described a theoretical perspective regarding the nature of mind-brain interaction involving what is termed the strong emergence hypothesis (SEH), in which an emergent self can function in a recurrent (or top-down) manner to integrate its mental properties and to rewire its brain. Among several clinically relevant examples of this empowering concept a cardinal one is the practice of mindful awareness, an observational practice that is enhanced by training, and that enables the self to modify or redirect attention onto objects that are deemed worthy of examination (7). Many studies have demonstrated an enhancement of frontal cortex function in conjunction with this practice (8). Of particular interest is a report by Hasenkamp et al. (9) showing that this mental training is capable of inducing the wandering of the mind to become a salient phenomenon (i.e., the wandering of the mind during the attempt to focus attention itself becomes a mental event that becomes salient and captures the attention). Furthermore, the process of taking notice of the wandering of attention and the subsequent re-focusing of attention is associated with a preferential activation of the brain's salience and executive networks in the insular and prefrontal cortices. However, there are numerous well-recognized psychological constructs that have not been linked to distinct neuronal pathways. In addition to clinically relevant and psychotherapeutically empowering concepts of confidence noted above, specific neuronal networks for “psychological denial,” “understanding context,” “ability to compartmentalize,” and analogous psychotherapeutically relevant mental states may well never be precisely mapped out. The main point is that while psychological phenomena are inseparable from brain functions, it has been extremely challenging to reduce them to brain mechanisms alone, especially in ways that are clinically applicable. This is largely due to the fact that the descriptions of the relationships between the respective physical and mental phenomena, as they are classified, measured and understood by currently available methodologies, reflect only statistical associations between imperfectly aligned terminologies, and certainly do not satisfactorily explain the causal relations between them. For instance, recent path analyses from a large naturalistic cohort show that genetic, brain imaging and psychological (e.g., self-reports) measures obtained at age 14 have an independent (instead of linear) contribution to the development of psychotic experience at age 18 (10). In other words, the anticipated relation of genetic vulnerability leading to abnormalities of brain function leading to altered self-reports of behavior leading to psychotic experiences was not observed, suggesting that molecular, physiological and psychological phenomena are somewhat independent. To restate the core aspect of our thesis, humility is called for by all concerned working at the interface of neuroscience and mental functioning.

This principle, i.e., that definitive brain involvement does not necessarily entail or imply clear-cut mechanisms of brain causation, has been, from the present authors perspective, reviewed in admirable detail in a recent work on the developmental neurobiology of fear learning and acquisition (11). In presenting a far-reaching theoretical perspective on how the processes involved in the formation of emotional bonds between caregiver and child come to guide maturation, Callaghan et al. propose a “developmental ecology” framework of fear neurobiology. This perspective leverages neuroconstructivist theories (12) that seek to explicate how the brain shapes itself into a specialized organ *via* developmentally critical goals that are expressed in the course of complex interactions with the environment. The effects of parenting on the development of emotional and stress regulation have been of particular interest in studies involving multiple mammalian species that are born requiring parental care for survival.

Specifically, a robust and growing body of research has aimed at characterizing the special modulatory/inhibitory, now frequently termed “buffering,” effects of parental- offspring interactions on fear-learning and stress-relevant neurobiology. This work has demonstrated, in both non-human and human subjects, that multiple physiological stress- responses can be significantly ameliorated by maternal presence, with effects in the amygdala and its connections with frontal cortical circuitry being particularly well-demonstrated and noteworthy (13). It is of great interest that even in rodents, as Sullivan and Perry (14) state, “the mother attenuates the neurobehavioral stress response in infancy and prevents pups from learning about threat within mother-infant interactions.” To a much more complex, intensified and of course, clinically significant degree, this effect has also been very well-demonstrated in humans, and decrements in the efficacy of this buffering, investigated in the context of early life adversity (manifested by disruptions in a child's relationship with caregiving figures), can occur even if caregivers are physically present [see (11) for review].

Such effects of early caregiving adversity have been well-demonstrated in animal models. In a recent report Raineki et al. (15) stated that, in rodents, “social context paired with stress hormones is required to produce amygdala-dependent social behavior deficits,” while explicitly stating that, especially in humans, many different aberrant developmental trajectories “are likely to coexist” and result in amygdala-dependent social behavior deficits.

From the perspective of the present authors, a key point to take from these extensive and highly sophisticated studies is that even though there is no question that the effects of caregiver and related forms of social buffering on physiological (and pathophysiological) stress-response and fear-learning neurobiology are extremely well-demonstrated, great care should still be taken in interpreting these very complex social effects on brain circuits and neurochemistry as resulting from any well-described (or, from our perspective, even necessarily describable) brain mechanisms. We certainly acknowledge that there are brain mechanisms that play a primary causal role in clinically significant disturbances of affect/mood, habit/craving, and repetitive patterns of thought/obsession (2). However, there are a wide variety of psychological and behavioral states which, even though also observed and modeled in other animals, including rodents, and even though these states are clearly associated with discrete neuroanatomical functional systems, do not have, and may never have, clearly delineated brain mechanisms that fully account for their complex experiential and clinical manifestations.

## Subjective Experience, Language, and the Brain

The notion that both biological and psychological processes form the base of mental disorders is hardly new. However, the prevailing paradigm seems to imply that there is a direct causal connection from biological to psychological phenomena, thus suggesting that biological systems and their functions are the ultimate cause of all psychological states. There is no dispute that the brain biological systems are a critical aspect of psychological phenomena. But it is too simplistic to suggest that the brain “creates” the mind in a way entirely analogous to how a nerve signal sent to an extremity will generate a muscle contraction. The analogy that the brain functions like a complex computer is of limited help as well — e.g., in his classic 1958 pamphlet The Computer and The Brain (16), his final publication, John von Neumann points out that, due to profound differences in the precision of connectivity between CNS neuronal synapses and “artificial computing machines” (p. 76) the languages used in brain computation must utilize “different logical structures from the ones we are ordinarily used to in logics and mathematics,” (p. 112) and thus are essentially different in kind from the languages of machines. While recent work (17, 18) has attempted to very creatively bridge these logical gaps between the brain and machines, we think all could agree that there is still a very long way to go to truly be able to assertively claim that a genuine translation between these radically different languages and precisions of intrinsic communication is even close to being accomplished. A key aspect of our perspective is that the work to bridge the gap between the logics of the computer and the brain very much remains a work in progress. Even more so, the clinical implications of that work, vis a vis the vast variety of both adaptive and pathological psychological states, is an area where collaborative efforts of people having a variety of sophisticated skill sets is necessary [as we have previously discussed in (19)].

This conundrum is further complicated by the lack of clarity as to what extent various psychological abilities are inherited vs. shaped by experience. In the current era the premise that there is a direct causal relationship between brain functions and psychological processes remains so strong that it is often assertively posited while remaining largely unquestioned. Furthermore, and very problematically from a clinical perspective, this premise of direct brain causality of psychological function is often seen as entailing the belief that if we devote adequate time, effort, and resources to fully understanding the functions of the brain, the puzzle of human behavior and its pathologies in all its forms will be solved.

One programmatic application of this premise of direct brain causality of psychological function is contained within the broad category of techniques often referred to as translational research: it posits that since all biological systems operate on similar principles, then “biological models” of human disease could be created in laboratory animals (or *in vitro* in cell or tissue cultures), and then manipulated by a variety of interventions, while assessing both biological and behavioral measures. This translation model has been used for various medical conditions—studies of antibiotics and vaccines heavily rely on it and have been very successful. It has been implemented in the studies of mental illness for well over two decades, alongside advances in genetics, structural and functional neuroimaging and more recently computational modeling. Together these novel methods of studying the brain have provided a wealth of information on brain morphology, physiology and modeling of behavior, yet while these data have exponentially increased in overall volume, the nature of the link between the biology of the brain and the psychology of the mind has remained elusive. As time has passed, the large community of clinically-oriented neuroscientists, psychologists and psychiatrists has begun to acknowledge that laboratory research and clinical practice seem to steadily drift ever further apart (20, 21).

As a result, some authors have begun to discuss the inherent limitations of these translational methods, most notably limitations linked to the fact that studying internal experiences and mental representations of the human condition through biological models in animal (or even *in vitro* human cellular) preparations leaves out one critically essential aspect of the clinical situation—verbal accounts of clinically relevant experiences that only a human can provide. For example, LeDoux et al. (22) have pointed out that contemporary approaches in mental health treatment (e.g., biological psychiatry and even traditional applications of cognitive behavioral therapy for instance) have often marginalized subjective well-being as an endpoint in treatment. Yet, if therapy does not result in patients both feeling and describing themselves as subjectively better, they are unlikely to feel that treatment was successful. The recognition of the value of verbal self-report is thus crucial to overcoming the limitations in the clinical application of translational methods. So too is the acknowledgment that translational methods are often intrinsically limited in the utilization of language by the very nature of studies of animal and cellular brain biology.

The importance of language for the assessment and monitoring of psychiatric conditions is primarily due to the following: first, humans are the only biological species capable of developing language in the form that is known to us. This is supported by the work of Herbert Terrence documenting that the apes that are genetically closest to humans lack capacity to understand semantics, syntax and grammar (23, 24). Therefore, human language conveys information that cannot be adequately assessed *via* any other methods that do not also use human language (which machine learning languages always require in the creation of codes, etc.). Second, the utility of verbal reports (as variable and even idiosyncratic they can be) has been documented in studies that aim to develop new medication treatments, and with heightened interest in human focused approaches possibly (though perhaps not advisedly) even the phasing out of animal modeling has been suggested (see editorial by Dr. Pandora Pound for special issue of *Animals* (2020, ISSN 2076-2615) (25).

We believe that, in principle, language is and remains an essential tool that clinicians must use in order to understand, diagnose and psychologically treat mental disorders (and it is very much worth emphasizing in this context that compliance with prescribed biological treatments is a critical aspect of psychological treatment). While it is of course true that language analyses have been used for years in developing and evaluating treatment interventions, and that there have been steady advances in the use brain imaging of to investigate how language and conversation/communication are related to neuroanatomical aspects of emotional responses to social stimuli, we must face the fact that we are still very far indeed from being able to biologically model concepts like “insight,” “implied meaning,” “understanding context,” and many other psychological states that clinicians critically rely on in everyday practice. And we are farther still from being able to characterize the clinical application of these kinds of psychological concepts in biological terms.

## The Clinical Relevance of the Interface of Mind and Brain

Significant advances have been made in modeling how the mind can create constructs of reality. For instance, as one keeps an image of another person in one's mind (i.e., not only images of physical features but the idea of that particular person as being friendly or unfriendly or something else) that mental representation will be superimposed and compared to the actual interpersonal interaction with the other person. The internal mental image may closely overlap or notably differ from the real-life experience/interaction and if so the mental representation may be adjusted accordingly.

So in psychological terms the mind operates in dyadic states—maintaining mental representations of individuals, events and so on—and comparing/adjusting these mental images to match the real-life experiences. If one uses computational modeling these processes can be analyzed and clarified with respect to a key factor known as prediction error—and recently a whole new direction of research has emerged aiming to understand behaviors in the context of preconceived notions (priors), registered outcomes (posteriors), and the comparisons of both (prediction error). This certainly could be a very fruitful direction since it has the potential to provide a theoretical and quantifiable link between patterns of neuron firing indexed *via* functional imaging, EEG, etc., and measures of task performance when both of these variables are analyzed within a Bayesian statistical framework (17). It is possible that such an approach can begin to bridge the gap between psychological symptoms and brain substrates. For instance, research has identified differences in the structure of the auditory cortex or its tonotopy in individuals who experience auditory hallucinations (26, 27). It is also possible to design tasks that evaluate auditory hallucinations and to model a participant's behavior on such tasks. Computational psychiatry has the tools to connect the activation patterns in the auditory cortex to subject's responses during the task in a coherent model ultimately showing where and how the cortex activates when a person reports auditory hallucinations. However, what would still remain out of the scope of such investigation are the effects that the experience of auditory hallucinations may have on the one's concept of self and their surrounding environment—presenting significant, but potentially rectifiable challenges that have been articulated in a recent publication (2). For example, while a diagnosis of schizophrenia might be established partially due to the presence of auditory hallucinations, the complex behaviors that may be linked to this symptom (paranoia, loosening of associations, difficulties in social functioning) will be far more difficult to predictively model. This difficulty is even more trenchant for concepts like one's self esteem and general perspectives on life as people internalize the idea that they suffer with a lifelong disorder with a profoundly unpredictable nature. And while new medications may be able to effectively suppress auditory hallucinations (or other symptoms), the painful and potentially disabling mental constructs that a person creates in their mind as a result of experiencing such symptoms for much of their lives will most likely remain unchanged as a result of drug administration alone. Of course, analogous patterns of potentially disabling patterns of cognition and pathological self-understanding will also be elicited by symptoms of affective, anxiety, substance use disorders, and so forth.

The development of psychological constructs that do not map onto neuronal networks is not merely a flaw of our thinking and a gap in our understanding—it rather reflects a reality that our thinking and understanding has not adequately adapted to. In a review focused on the understanding and treatment of anxiety LeDoux and Pine (1) state that the development of efficacious psychotropic agents is hampered by “two faulty assumptions: (1) that a common circuit underlies the expression of defensive responses and feelings of fear when threatened, and (2) that the circuits that contribute to defensive responses in animals can be used to determine how the human brain gives rise to feelings of fear and anxiety.” The key point here is to differentiate between neurocircuits that may produce the expression of physiological (hyperarousal) and behavioral (avoidance) manifestations of anxiety (e.g., symptoms of tachycardia, muscular tension, and “freezing”) and the construct of fear, which includes the subjective experience of anxiety, and is generated through the engagement of the second order psychological processes that create mental representations in one's mind—and to be aware that the latter may not be readily reducible solely to the activity of neuronal circuits. These mental representations are consciously experienced and often become “ego-congruent,” which in psychiatric terms refers to experiences and beliefs that are in synchrony and consistent with one's sense of self. It is very probable that individuals who go on to develop mental illness may show brain activation and connectivity in particular brain networks that are different from the activation and connectivity in corresponding brain systems of individuals who enjoy mental “normalcy.” However, the clinical effects of those brain differences will be amplified and exacerbated by psychological constructs, elicited by those brain systems that have been developed over time as one encounters a wide variety of stimuli in the course of one's life experience. These differences in psychological constructs (i.e., the outside world is a relatively safe place vs. it is a dangerous and aversive) are probably significantly less susceptible to the manipulations of receptor systems and patterns of activity in particular brain regions. The key point is that although the psychological constructs formed in response to physical symptoms of anxiety are certainly influenced by pathological changes in neural circuitry, there is no direct correlation between “normalization” of the brain circuitry underlying physical symptoms and the alleviation of functional deficits caused by the psychological constructs associated with anxiety disorders. One can speculate that in psychological terms the mind has “internalized” pathology into one's sense of self, and this basic function—to recognize patterns of repetition and sameness and to categorize them as “myself” —tends to persist in a manner that has been established over lengthy periods of time. The complexity and clinical importance of these biological-psychological relationships, as well as the capacity of computational approaches to discern clinically relevant aspects of their interaction, has very recently been nicely demonstrated by Berwian et al. (28) in subjects with remitted depression who were still being treated with antidepressant medications. They found that even while their depression is in remission, patients taking antidepressant medications show performance differences on a task requiring participants to choose how much effort to exert for various amounts of reward, and that these differences might be a clinically useful predictor of relapse if medication is discontinued.

Based on this it seems reasonable to suggest that the role of pharmacological agents in psychiatry may be best defined as tools for the amelioration of symptoms that can further create conditions for other psychologically-oriented therapeutic modalities (e.g., cognitive therapy) to take hold; and perhaps to add that due to the very nature of the dichotomy between biological and psychological processes with respect to the emergence of mental illness, it is unrealistic to expect that personal beliefs about one's self and environment may dependably be crucially altered and adaptively “normalized” by the administration of medication(s) (and/or other physical interventions) alone.

To summarize, our attempt to make translational research more genuinely clinically relevant focuses on the unavoidably integrative nature of all mental health interventions that are likely to be reliably and reproducibly effective in actual clinical practice—our approach might best be understood as a radically updated revision of the bio-psycho- social models of prior eras. While we certainly believe that advances in the collection and statistical analysis of “big data” packages using machine learning and related techniques are important and need to be encouraged, we also think that clinical improvements in the understanding and prediction of the emergence, progression and treatment of mental illnesses are not likely to be significantly advanced merely by the addition of new outcome measures related solely to drug response and efficacy. And while we certainly agree that laboratory measures such as gene expression and hormonal activity may well be extremely helpful in understanding and predicting syndromal/disease vulnerability and symptom expression, they are not likely be genuinely diagnostic when used in isolation. Our field needs to continue its efforts to understand how psychiatric disorders are linked to functional networks of genetic, neuroanatomical and environmental interactions, as opposed to thinking that investigating a gene, or a set of genes, or distinct brain structures in isolation can usefully advance clinically relevant knowledge. Novel approaches such as integrating drugs that can ameliorate a variety of symptoms with individualized cognitive-behavioral and related psychological treatments are coming close to FDA approval as effective treatments for complex conditions like PTSD (29). Additional elements in these kinds of integrated approaches would be the use of portable brain imaging technologies such as near infrared spectroscopy (fNIRS) imaging, imaging guided biofeedback, cognitive exercise video programs, electronic self-assessment tools and reminders, virtual reality trainings, mindfulness training, quicker access to care *via* telehealth, etc. Very importantly, using telehealth to focus on the synergistic integration of biological and psychological treatments has the potential to expand person-to-person interventions to locales outside of therapists' offices, and gives clinicians the opportunity to make more detailed assessments of their patients' daily real-world physical, social and mental environments. The application of the medical model to psychiatry remains important, and we believe it can be enhanced by a proper understanding of its limitations. Deeper insights into how psychosocial factors influence biological mechanisms will enable our efforts to integrate biological and psychological interventions in ways that more effectively change the course of mental illness and create significant additional benefits, particularly with respect to issues of enhanced self-efficacy among those who suffer from mental health problems. We want to state categorically that we view enhanced self- efficacy (by which we mean effective self-regulation and self-management when coping with stress-inducing changes in bio-psycho-social factors) as one of the primary goals of all clinical interventions in the fields of mental health treatment, and that integrative approaches are necessary to achieving that goal. Ongoing integrations of the biological/medical model with cognitive-behavioral/mindfulness-based models, especially in investigations of more invasive and/or novel treatment modalities (e.g., intravenous medications like ketamine for refractory depression, or deep brain stimulation for OCD, or the use of psychedelics) can provide a greater promise for long- term benefits and improved functioning in treating otherwise refractory clinical syndromes, while synergistically enhancing the psychological benefits of existing biomedical interventions.

## Data Availability Statement

The original contributions generated for the study are included in the article/supplementary material, further inquiries can be directed to the corresponding author/s.

## Author Contributions

All authors listed have made a substantial, direct and intellectual contribution to the work, and approved it for publication.

## Conflict of Interest

The authors declare that the research was conducted in the absence of any commercial or financial relationships that could be construed as a potential conflict of interest.
